# Prevalence and Factors Related to Pathological Laughter and Crying in Patients With Amyotrophic Lateral Sclerosis

**DOI:** 10.3389/fneur.2021.655674

**Published:** 2021-09-28

**Authors:** Qian-Qian Wei, Ruwei Ou, Junyu Lin, Lingyu Zhang, Yanbing Hou, Bei Cao, Yongping Chen, Tianmi Yang, Huifang Shang

**Affiliations:** Department of Neurology, Laboratory of Neurodegenerative Disorders, West China Hospital, Sichuan University, Chengdu, China

**Keywords:** amyotrophic lateral sclerosis, pathological laughter and crying, depression, anxiety, cognition, causal effect

## Abstract

**Objective:** This study aimed to explore the prevalence and clinical correlates of pathological laughter and crying (PLC) in patients with amyotrophic lateral sclerosis (ALS).

**Methods:** A total of 1,031 ALS patients were enrolled between August 2012 and August 2019. The PLC was recorded by a face-to-face interview. Other characteristics of patients, including depression, anxiety, cognition, and behavior function, were also evaluated. The potential associated factors of PLC were explored using forward binary regression analysis. Survival was analyzed in groups using propensity score matching (PSM) and Cox proportional hazards models.

**Results:** The prevalence of PLC was 11.4% in all patients at baseline. Bulbar-onset and female patients had higher prevalence of PLC. The multivariate regression analysis indicated that PLC in ALS was associated with bulbar onset (*p* < 0.001), late disease stage (*p* < 0.001), and higher score in the Hamilton Depression Rating Scale (HDRS) (*p* = 0.012). The higher score of HDRS was significantly and independently associated with PLC occurrence in bulbar-onset patients (*p* = 0.032). The late disease stage was related to PLC occurrence in spinal-onset patients (*p* < 0.001). After comparison with matched pairs by using PSM, PLC at baseline had no impact on survival.

**Conclusion:** PLC was not uncommon in ALS, especially in bulbar-onset and female patients. We highlighted that the emotional state other than cognitive function had possible relationship with PLC in ALS.

## Introduction

Pathological laughing and crying (PLC) is a neuropsychiatric condition that patients demonstrated exaggerated or inappropriate episodes of laughter, crying, or both, in the absence of an apparent motivating stimulus or in response to stimuli that would not usually elicit such an emotional response (1). Various terms are used to referring to the PLC, depending on geographical location and year of publication, including emotional lability, pseudobulbar affect, involuntary emotional expression disorder, and so on. Despite the differences in terminology, the phenomenon has been observed in many neurological conditions including amyotrophic lateral sclerosis (ALS) (1, 2).

Previous research confirmed the PLC prevalence in different subgroups stratified by clinical features in ALS. A recent study reported that the prevalence of PLC varied across the spectrum of disease phenotypes, for instance, 39% in primary lateral sclerosis and upper motor neuron predominant ALS, 29% in typical ALS, and 10% in lower motor neuron predominant patients (3). PLC is negatively affecting the social functioning and quality of life of patients and their families (4, 5). Such unscheduled episodes may contribute to significant distress and embarrassment and ultimately may result in social withdrawal (6). ALS patients and their families usually lack awareness of the relationships between PLC and their underlying neurological condition. Thus, it was important to explore the PLC symptoms in ALS patients with high-risk conditions (7).

There were limited studies on the motor and non-motor features associated with PLC in ALS patients. PLC was found to be associated with female, bulbar onset, lower ALS Functional Rating Scale–Revised (ALSFRS-R) score, and more rapidly progressive disease rate in univariate analysis (3). Few studies have reported the relationships among PLC, emotional state, and cognitive function. A study detected an association between PLC and depression (8), whereas no association was found in another research (9). Cognitive dysfunction is a non-motor symptom in ALS with the severity of cognitive decline ranging from mild cognitive impairment to ALS frontotemporal dementia involving executive and non-executive domains (10). The relationship between PLC and cognitive impairment is controversial in ALS (11). Further studies with a larger sample size are required to explore the individualized experiences of PLC in ALS.

Therefore, the present study aimed to examine the prevalence of PLC in ALS in a large cohort. We also explored the relationships among PLC, emotional state, and cognitive function. Finally, we explored their prognostic relevance to survival.

## Patients and Methods

The study was conducted in our tertiary referral center for motor neuron disease in southwest China (Department of Neurology, West China Hospital of Sichuan University, Chengdu, Sichuan province) between August 2012 and August 2019. Definite, probable, and possible ALS cases diagnosed according to the El Escorial revised criteria were included. Patients with possible ALS at the time of recruitment were reclassified to a higher El Escorial level during the follow-up. The information of PLC was collected from the patients and their caregivers in face-to-face interviews. The PLC was diagnosed by the neurologists according to the revised criteria proposed by Cummings et al. (12) and Miller et al. (13). Information for the demographic features and personal history was recorded through questionnaires. Disease-related data were collected, such as age at onset, the region of symptom onset (upper limb, lower limb, or bulbar), disease duration, and diagnostic delay. Patients with familial ALS or juvenile ALS or who had incomplete data were excluded. Patients who were younger than 45 years at the age at onset were classified into the young-onset ALS group. The changes of ALSFRS-R per month [formula: (48-ALSFRS-R score at the baseline)/month intervals between the first symptom onset and baseline] were used to calculate the rate of disease progression. Disease stages according to King's College Staging System were recorded. Patients in stage 1 or stage 2 were divided into early-stage subgroup, and patients in stage 3 or stage 4 were divided into late-stage subgroup. Depression was evaluated by the 24-item Hamilton Depression Rating Scale (HDRS) (14), and anxiety was evaluated by the Hamilton Anxiety Rating Scale (HARS) (15). An HDRS score >20 indicated depression, and an HARS score >14 indicated anxiety. Frontal assessment battery (FAB) was used to assess the frontal lobe executive function (16). The FAB score <16 was defined as executive dysfunction according to our previous study (17). The global cognition function was evaluated by the Montreal Cognitive Assessment (MoCA) (18). The Chinese version of Addenbrooke’s Cognitive Examination–Revised (ACE-R) was adopted to evaluate further spectrum cognitive function in ALS patients. Higher ACE-R scores indicated better cognitive function in ALS. The ACE-R score of <75 was defined as cognitive dysfunction according to our previous study (19). Frontal behavioral symptoms were assessed using the Frontal Behavioral Inventory (FBI). Lower scores indicated better frontal behavioral function.

All ALS patients were followed up by telephone or face-to-face interview in 3- or 6-month intervals by neurologists (Q.Q.W., L.Y.Z., and Y.B.H.). All the clinical and treatment data, including disease progression, use of riluzole, and supportive treatment, were collected during the follow-up evaluations. Death information was collected from provincial public security bureau records and family reports. Survival time was defined as the interval time from the date of disease onset to the date of death for the deceased patients or from disease onset to the last follow-up time for survival patients. All the patients were informed of this study and signed written informed consent. This study was approval by the Ethics Committee of West China Hospital of Sichuan University.

### Statistical Analysis

First, we used the Kolmogorov–Smirnov test to determine whether data were normally distributed. Continuous parameters were described as the mean ± standard deviation when they were normally distributed. Those with a non-normal distribution were described as the median values. Continuous variables were compared using Student *t*-tests between subgroups. Mann–Whitney *U*-tests were implemented to analyze the difference between categorical variables. Subgroup analyses were conducted regarding onset region. The variables would be further included as independent variables in the binary regression analysis to explore the potential associated factors of PLC. The presence or absence of PLC was used as a dependent variable. To explore the impact of PLC on survival, we applied the propensity score matching (PSM) analysis to decrease the effect of potential confounding factors. One-to-one matching was performed based on nearest-neighbor matching adjusting onset age and disease progressive rate, and the match tolerance for the PSM was 0.1. The resulting propensity score–matched pairs (defined as the PLC group and the matched-pairs group) were included in subsequent survival analyses. The Kaplan–Meier curves and log-rank tests were used, and survival calculated from disease onset to death or the censoring date (January 2020). Multivariable analysis was performed by Cox proportional hazards regression model to explore the effect of PLC on survival (stepwise forward), with a retention criterion of *p* < 0.1. The hazard ratios and their 95% confidence intervals were calculated (20). The interactions between parameters were tested in the study. A level of *p* < 0.05 was considered statistically significant. All analyses were performed with SPSS 26.0 (SPSS, Inc., Chicago, IL, USA).

## Results

Demographic and clinical features of ALS patients with and without PLC according to different onset regions are shown in Table 1. In 1,031 ALS patients, the mean onset age was 53.1 ± 11.1 years, and the mean disease duration was 14.8 ± 11.3 months at baseline, respectively. Two hundred forty-four patients (23.7%) were young onset. One hundred fifty-eight patients (15.3%) were bulbar onset, and 84.7% of the patients were spinal onset. The mean disease progression rate for patients at register was 0.68 ± 0.65. All patients completed the anxiety, depression, and cognition assessment. The mean HDRS score was 9.0 ± 7.3, and the mean HARS score was 5.6 ± 5.9. According to the FAB score, 28.5% of patients had abnormal frontal function, and 27.7% of patients had cognitive decline according to the ACE-R score. The mean MoCA score was 23.8 ± 4.1, and the mean FBI score was 4.5 ± 6.5. PLC was diagnosed in 118 patients (11.4%) with ALS. The prevalence of PLC was 32.9% in bulbar-onset patients, and 7.6% in spinal-onset patients. Female patients had higher prevalence of PLC (14.6%) than male patients (9.6%).

**Table 1:** Demographic and clinical features of ALS patients with and without PLC.

**Variables**	**Total**	**Bulbar onset**			**Spinal onset**		
		**With PLC**	**Without PLC**	***P*** **-value**	**With PLC**	**Without PLC**	***P*** **-value**
Number	1,031	52 (32.9%)	106 (67.1%)		66 (7.6%)	807 (92.4%)	
Age (y)	54.3 ± 11.1	56.0 ± 11.1	57.7 ± 10.1	0.337	51.3 ± 10.1	54.0 ± 11.2	0.037^*^
Sex (male, %)	63.5	50.0	52.8	0.738	56.1	66.4	0.088
Education (y)	9.6 ± 3.4	9.9 ± 3.6	9.1 ± 3.5	0.241	8.9 ± 3.4	9.6 ± 3.4	0.100
Smoking (never, %)	54.4	59.6	60.4	0.765	62.1	52.8	0.340
Drinking (never, %)	64.5	67.3	67.9	0.121	60.6	64.3	0.740
BMI	22.0 ± 2.9	21.5 ± 2.8	21.8 ± 3.3	0.649	22.6 ± 3.0	22.0 ± 2.9	0.089
Onset age (y)	53.1 ± 11.1	54.9 ± 11.1	56.7 ± 10.1	0.301	50.0 ± 10.1	52.8 ± 11.2	0.037^*^
Young-onset (%)	23.7	19.2	12.3	0.243	24.2	25.4	0.835
Diagnostic duration (m)	14.8 ± 11.3	13.4 ± 11.8	11.9 ± 11.4	0.437	15.2 ± 12.6	15.3 ± 11.1	0.950
Diagnostic delay (m)	13.7 ± 10.7	11.7 ± 9.2	11.2 ± 11.2	0.784	13.5 ± 11.7	14.2 ± 10.6	0.630
ALSFRS-R score	40.9 ± 4.8	40.7 ± 6.0	42.6 ± 3.9	0.016^*^	37.7 ± 5.0	41.0 ± 4.6	<0.001^*^
Disease progression rate	0.68 ± 0.65	0.78 ± 0.94	0.68 ± 0.65	0.451	0.95 ± 0.64	0.65 ± 0.62	<0.001^*^
Early stages (%)	71.8	55.8	44.2	0.169	39.4	76.1	<0.001^*^
Gene mutations	158/569	11/34	15/60	0.444	14/37	118/438	0.155
Using riluzole (%)	36.4	50.0	43.4	0.434	37.9	34.4	0.574

*ALS, amyotrophic lateral sclerosis; ALSFRS-R, ALS functional rating scale revised; PLC, pathological laughter and crying; BMI, body mass index*.

* *Significant difference*.

In the bulbar-onset group, a lower ALSFRS-R score (40.7 ± 6.0 vs. 42.6 ± 3.9, *p* = 0.016) was found in patients with PLC compared with those without. In the spinal-onset group, compared with patients without PLC, patients with PLC were younger (*p* = 0.037) and had younger age at onset (*p* = 0.037), lower ALSFRS-R score (*p* < 0.001), higher disease progression rate (*p* < 0.001), and lower percentage of early stages (*p* < 0.001; Table 1). The cognitive characteristics between ALS patients with and without PLC are demonstrated in Table 2. In the bulbar-onset subgroup, there was no difference in the cognitive features between patients with and without PLC. In the spinal-onset subgroup, compared with patients without PLC, patients with PLC had higher HDRS score (*p* = 0.011), lower MoCA score (*p* = 0.024), lower visuospatial ability score (*p* = 0.008), lower ACE-R total score (*p* = 0.030), and higher percentage of cognitive decline according to ACE-R score (*p* = 0.017; Table 2).

**Table 2:** Mood and cognitive function of ALS patients with and without PLC.

**Variables**	**Total**	**Bulbar onset**			**Spinal onset**		
		**With PLC**	**Without PLC**	***P*** **-value**	**With PLC**	**Without PLC**	***P*** **-value**
HDRS score	9.0 ± 7.3	9.7 ± 7.0	7.6 ± 6.7	0.067	11.3 ± 7.9	8.9 ± 7.3	0.011^*^
HARS score	5.6 ± 5.9	5.6 ± 5.4	5.3 ± 5.3	0.709	6.9 ± 6.5	5.5 ± 5.9	0.068
FAB	16.2 ± 2.1	16.3 ± 1.6	15.8 ± 2.3	0.214	15.8 ± 2.7	16.2 ± 2.0	0.092
FAB <16 (%)	28.5	30.8	35.8	0.527	34.8	26.9	0.164
FBI score	4.5 ± 6.5	6.4 ± 8.2	5.3 ± 6.8	0.466	5.5 ± 8.4	4.1 ± 6.1	0.162
MoCA	23.8 ± 4.1	23.7 ± 4.0	23.4 ± 4.1	0.672	22.8 ± 4.6	24.0 ± 4.0	0.024^*^
**ACE-R**							
Attention/orientation	17.0 ± 1.5	16.9 ± 1.4	17.0 ± 1.2	0.659	16.7 ± 1.3	17.0 ± 1.6	0.196
Memory	21.2 ± 4.4	21.6 ± 3.7	21.2 ± 4.7	0.548	20.6 ± 4.6	21.2 ± 4.4	0.299
Verbal fluency	9.2 ± 2.5	8.3 ± 3.5	9.0 ± 2.7	0.210	8.7 ± 2.4	9.4 ± 2.4	0.042
Language	19.6 ± 4.6	18.6 ± 4.9	19.2 ± 4.6	0.487	19.0 ± 4.9	19.8 ± 4.5	0.154
Visuospatial ability	13.4 ± 2.9	13.1 ± 2.8	13.2 ± 2.8	0.785	12.4 ± 3.5	13.6 ± 2.8	0.008^*^
ACE-R total score	80.5 ± 12.0	78.6 ± 12.6	79.7 ± 12.1	0.603	77.7 ± 13.3	81.0 ± 11.9	0.030^*^
ACE-R <75 (%)	27.7	36.5	30.2	0.422	39.4	25.9	0.017^*^

*ALS, amyotrophic lateral sclerosis; PLC, pathological laughter and crying; HDRS, Hamilton depression rating scale; HARS, Hamilton anxiety rating scale; FAB, frontal assessment battery; FBI, Frontal behavioral inventory; MoCA, Montreal cognitive assessment; ACE-R, Addenbrooke's cognitive examination-revised*.

* *Significant difference*.

The potential factors associated with PLC in ALS patients are shown in Table 3. The logistic regression model indicated that PLC was significantly associated with bulbar onset (*p* < 0.001), late disease stages (*p* < 0.001), and higher score of HDRS (*p* = 0.008). Higher score of HDRS (*p* = 0.032) was associated with PLC in the bulbar-onset group. Late disease stage (*p* < 0.001) was significantly and independently related to PLC occurrence in patients with spinal onset (Table 3).

**Table 3:** Factors associated with PLC in ALS patients with different onset region.

**Variables**	**All patients**			**Bulbar onset patients**			**Spinal onset patients**		
	**OR**	**95% CI**	***P*** **-value**	**OR**	**95% CI**	***P*** **-value**	**OR**	**95% CI**	***P*** **-value**
Education							0.920	0.837–1.012	0.086
Depression	1.069	1.015–1.126	0.012^*^	1.117	1.009–1.235	0.032^*^			
Anxiety	0.941	0.877–1.010	0.091	0.887	0.772–1.019	0.091			
Early stages	3.211	1.958–5.267	<0.001^*^				6.205	3.263–11.799	<0.001^*^
Onset region	0.159	0.095–0.267	<0.001^*^						
ACE-R	0.648	0.389–1.077	0.094						

* *Significant difference*.

*OR, hazard ratios; CIs, confidence intervals; ACE-R, Addenbrooke's cognitive examination-revised*.

At the end of the follow-up, 461 ALS patients (44.7%) died, 535 patients (51.9%) were alive, and 35 patients (3.4%) were lost to follow-up. For all patients, the mean survival time was 36.1 ± 22.7 months. In the present study, 36.4% of the patients were using riluzole. In all patients, the Kaplan–Meier survival analysis indicated that the survival time has no significantly difference between the groups (log-rank *p* = 0.098) (estimated median survival time: 38.0 vs. 49.7 months).

PSM was applied to adjust the potential confounding factors on the survival in ALS patients. As shown in Table 4, 116 patients were in the PLC group and 116 patients in the matched-pairs group. The onset age, ALSFRS-R score, and disease progression rate were comparable between the two groups. Patients with PLC had higher percentage of late disease stage than patients without PLC. In the subgroup analysis, after comparing the matched pairs, there was no significant difference in the survival time between patients with and without PLC (log-rank *p* > 0.05). In the multivariate Cox regression analysis, PLC had no significant effect on survival after adjusting for baseline clinical features, HDRS, HARS, cognitive function, and treatment.

**Table 4:** Comparison of cognitive features at baseline between patients and matched pairs groups.

**Variables**	**Bulbar onset**			**Spinal onset**		
	**With PLC**	**Without PLC**	***P*** **-value**	**With PLC**	**Without PLC**	***P*** **-value**
Number	50	50		66	66	
Onset age (y)	54.9 ± 11.2	55.3 ± 10.5	0.853	50.0 ± 10.1	51.7 ± 11.7	0.374
ALSFRS-R score	41.5 ± 4.6	42.2 ± 4.3	0.382	37.7 ± 5.0	39.1 ± 6.0	0.157
Disease progression rate	0.78 ± 0.96	0.74 ± 0.71	0.799	0.95 ± 0.64	0.77 ± 0.71	0.133
Early stages	29	21	0.839	26	40	0.015^*^
HDRS score	9.9 ± 7.0	8.9 ± 7.9	0.527	11.3 ± 7.9	8.6 ± 7.7	0.054
HARS score	5.7 ± 5.4	5.7 ± 5.7	0.966	6.9 ± 6.5	5.1 ± 6.1	0.107
FAB	16.2 ± 1.6	15.9 ± 2.2	0.433	15.8 ± 2.7	16.1 ± 2.1	0.476
FBI score	6.3 ± 8.4	4.4 ± 4.7	0.224	5.5 ± 8.4	2.7 ± 5.7	0.059
MOCA	23.6 ± 4.0	23.2 ± 3.9	0.661	22.8 ± 4.6	23.0 ± 4.4	0.839
**ACE-R**						
Attention/orientation	16.9 ± 1.4	17.0 ± 1.2	0.759	16.7 ± 1.3	16.7 ± 1.7	0.821
Memory	21.7 ± 3.7	21.3 ± 5.0	0.618	20.6 ± 4.6	21.9 ± 4.0	0.102
Verbal fluency	8.3 ± 3.6	8.6 ± 2.9	0.669	8.7 ± 2.4	9.2 ± 2.8	0.258
Language	18.6 ± 4.9	19.1 ± 4.5	0.611	19.0 ± 4.9	18.6 ± 4.8	0.640
Visuospatial ability	13.1 ± 2.7	13.2 ± 2.7	0.855	12.4 ± 3.5	12.3 ± 3.4	0.919
ACE-R score	78.6 ± 12.6	79.1 ± 12.1	0.840	77.7 ± 13.3	78.7 ± 13.3	0.667

*PLC, pathological laughter and crying; ALSFRS-R, ALS functional rating scale revised; HDRS, Hamilton depression rating scale; HARS, Hamilton anxiety rating scale; FAB, frontal assessment battery; FBI, Frontal behavioral inventory; MoCA, Montreal cognitive assessment; ACE-R, Addenbrooke's cognitive examination-revised*.

* *Significant difference*.

## Discussion

In the present study, we explored the prevalence and the associated factors of PLC in a large cohort of Chinese ALS patients. We found that the PLC was not uncommon in Chinese ALS patients. A higher prevalence of PLC was identified in bulbar-onset and female patients with ALS. Bulbar-onset and late disease stages were associated with PLC in ALS. We also highlighted that the emotional state other than cognitive function had possible relationships with PLC in ALS.

The frequency of PLC has been examined in many neurodegenerative diseases, which is particularly common in ALS. We found that PLC occurred in 11.4% of our registered patients. The prevalence is considerably lower than 28.4% from an unselected outpatient population with ALS (3), and 34% from a population-based study using the Center for Neurologic Study–Lability Scale (CNS-LS) (21). Another cross-sectional study reported a higher frequency of 45% in ALS patients (22). It is difficult for clinicians to recognize PLC in ALS patients unless a formal or structured interview is performed, and/or they have been directly observed. A standardized self-assessment CNS-LS was used in these studies [3, 21. 22], while our study evaluated PLC clinically during face-to-face interviews. The sample size and the heterogeneity in patients, such as the proportion of patients with bulbar onset and varied disease duration, may also influence the frequency of PLC in ALS. Our study confirmed the previously described and clinically recognized associations between onset region and PLC in ALS. The prevalence of PLC was 32.9% in bulbar-onset patients, which is higher than 7.6% in spinal-onset patients. This was consistent with a previous study, which found that almost 42.6% of the bulbar-onset patients had PLC, whereas 23.3% of the non–bulbar-onset patients had PLC (3).

Little attention was paid to the association between sex and PLC. Two previous studies found a weak trend that female patients with ALS had higher frequency of PLC than male patients (21, 22). This association was confirmed by another study (37.8 vs. 21.4%) (3) and the current study (14.6 vs. 9.6%). Female patients with other neurological conditions are more likely to have emotional lability, particularly pathological crying. There may be sex-specific background or pathology underlying the different prevalence of PLC. Altogether, PLC is not rare in ALS, and neurologists should pay more attention to this problem, especially in bulbar-onset and female patients.

In the spinal-onset group, we found that patients with PLC had younger age at onset, lower ALSFRS-R score, and higher disease progression rate. ALS patients with PLC also had higher HDRS score and lower ACE-R score than patients without PLC. The mood state and cognitive function seemed to relate to the development of PLC in ALS patients. PLC and depression have been found to coexist more frequently than expected by chance in other diseases in previous studies (23, 24). Thus, the interaction of pseudobulbar affect and depression is complex. Some studies reported an association between PLC and depression in ALS (8), whereas no significant association was found in others (9, 25). In our regression model, the depression evaluated by HDRS was found to be associated with the presence of PLC in ALS patients.

The PLC and cognitive function were negatively affecting the social functioning and quality of life in ALS individuals and their families (4, 5, 26, 27). The PLC symptoms were keenly awarded by patients and caregivers (28). While patients' reports may differ substantially from caregivers' reports in cognitive dysfunction or behavioral deficits (29), there is little information about the possible relationships of these phenomena. A previous study reported correlations between bulbar involvement and emotional lability and between bulbar involvement and executive impairment. However, other cognitive profiles have not been reported to correlate with PLC (28). In this study, cognitive dysfunction was not associated with the presence of PLC in ALS using a multivariate regression model.

Recent studies suggested that the dysfunction of the cerebellum was involved in the pathophysiology of PLC. Studies indicated that the cerebellum may regulate the emotional output through a “gate-control” mechanism (30). Several studies focused on neurological diseases have confirmed the association between PLC and cerebellar pathology, especially linked with vermis pathology. The high prevalence of PLC in multiple system atrophy cerebellar type patients with severe structural atrophy of the cerebellum and basis pontis provided evidence for this potential mechanism (31). The gate control theory of emotional expression, indicating that the disruption to corticopontocerebellar emotional circuitry may underlie this phenomenon. On the other hand, serotonergic and glutamatergic neurotransmission may play important roles in the pathophysiology of PLC (13). The study indicates that serotonin may be involved in the pathophysiology of PLC through the diffuse corticolimbic networks participating in emotion or *via* serotonergic neurotransmission in the cerebellum (13). PLC may be relieved by selective serotonin reuptake inhibitors within days. The effective treatment of PLC remains challenging; thus, studies should pay more attention to the treatment of PLC in patients with ALS.

We failed to observe a significant impact of PLC on survival in ALS patients in a multivariate Cox regression model analysis. A previous study found that the presence of PLC at recruitment can be a negative prognostic factor for survival in a very early stage of the disease, suggesting a short survival in a subgroup of patients with less functional impairment (11). However, this study lacked the neuropsychological evaluation for the relationship between PLC and other symptoms such as frontal dysfunction or cognitive impairment in general. The depression and anxiety were not assessed in this study also. Therefore, the conclusion that PLC can influence the prognosis is not convinced, and further studies are needed to confirm it.

Several limitations of this study should be noted. We only estimated the presence of PLC from a face-to-face interview, and it might be more comprehensive in case we use a specific scale to assess the severity of PLC, such as CNS-LS, in a structured interview (3). Neuroimaging data are helpful to explore the mechanism of PLC, which need to be considered in future studies. A potential sample selection bias may affect our results as all cases were recruited solely in a single tertiary referral center. A previous study reported that crying-predominant PLC was often associated with depression, whereas PLC with laughter-predominant PLC was not (3), although our study did not classify PLC into laughter-predominant or crying-predominant subgroups.

## Conclusion

Our study demonstrated that PLC was not uncommon in ALS, especially in bulbar-onset and female patients. We highlighted that the emotional state other than cognitive function had a possible causal effect on PLC in ALS. Further studies are needed to confirm the preliminary findings.

## Data Availability Statement

The raw data supporting the conclusions of this article will be made available by the authors, without undue reservation.

## Ethics Statement

The studies involving human participants were reviewed and approved by the Ethics Committee of West China Hospital of Sichuan University. The patients/participants provided their written informed consent to participate in this study.

## Author Contributions

Q-QW contributed with conception, organization and execution, data collection and statistical analysis, and drafting the manuscript. RO contributed with execution, data collection, and statistical analysis. JL, LZ, YH, YC, and TY contributed with execution and data collection. BC contributed with conception, organization, execution, and data collection. HS contributed with conception and organization, manuscript review and critique, and responsible for overall content as the guarantor. All authors contributed to the article and approved the submitted version.

## Funding

This study was supported by the funds of the Sichuan Science and Technology Program (Grant No. 2020YFS0220), National Science Fund of China (Grant No. 81871000), 1.3.5 project for disciplines of excellence, West China Hospital, Sichuan University (Grant No. ZYJC18038), the China Post-doctoral Science Foundation (Grant No. 2019M653427), Post-Doctor Research Project, West China Hospital, Sichuan University (Grant No. 2019HXBH029), and Health Commission of Sichuan Province (Grant No. 20PJ038).

## Conflict of Interest

The authors declare that the research was conducted in the absence of any commercial or financial relationships that could be construed as a potential conflict of interest.

## Publisher's Note

All claims expressed in this article are solely those of the authors and do not necessarily represent those of their affiliated organizations, or those of the publisher, the editors and the reviewers. Any product that may be evaluated in this article, or claim that may be made by its manufacturer, is not guaranteed or endorsed by the publisher.
